# Plastid translation as a developmental checkpoint? Plastid ribosomal protein EMB27 is required for maize embryogenesis

**DOI:** 10.1093/plphys/kiae106

**Published:** 2024-02-24

**Authors:** Janlo M Robil

**Affiliations:** Assistant Features Editor, Plant Physiology, American Society of Plant Biologists; Department of Biology, School of Science and Engineering, Ateneo de Manila University, Quezon City 1108, Philippines

Plant embryos undergo crucial development to establish themselves for photosynthesis. It begins at the early stages of embryogenesis with the formation of plastids in cells of nascent ground and epidermal tissues (West and Harada 1993). Genetic screens in Arabidopsis and maize have revealed embryo lethality in numerous chloroplast-defective mutants (Meinke 2020). However, the observed phenotypic variations across different species and genetic backgrounds complicate our understanding of the relationship between these 2 defects (Parker et al. 2014). Therefore, how chloroplast biogenesis influences plant embryogenesis remains unclear.

In this issue of *Plant Physiology*, Liu et al. (2024) explore the roles of EMB27, a plastid ribosomal protein S13 (RPS13), in maize development. Through genetic experiments and biochemical assays, the authors provide evidence that EMB27 is involved in plastid protein translation, required in both plastid and embryo development (Fig. 1A). The functional analysis of EMB27 offers some insights into the yet-to-be-resolved link between chloroplast biogenesis and plant embryogenesis.

**Figure 1.**
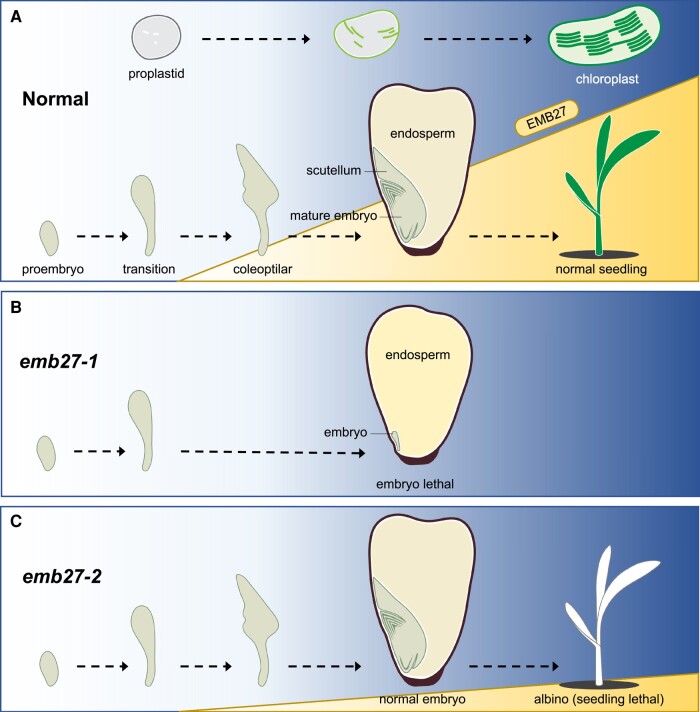
The role of EMBRYO DEFECTIVE27 (EMB27) in chloroplast biogenesis and embryogenesis in maize. **A)** A schematic diagram illustrating chloroplast biogenesis (top) and maize embryo development (bottom). Early maize embryo development is characterized by the proembryo, transition, and coleoptilar stages. EMB27 is progressively required for both embryo development and seedling pigmentation. The yellow ramp represents the increasing expression levels of *EMB27*. **B)** The null mutant *emb27-1* displays an arrest in embryo development at the transition stage, while endosperm development remains normal. **C)** The *emb27-2* mutant, which exhibits partial *EMB27* expression, shows normal embryo development but fails to form functional chloroplasts, resulting in albinism and seedling lethality.

The investigation started with the isolation of the *embryo defective27-1* (*emb27-1*) mutant from a transposon insertion population. A mature maize kernel is composed of an embryo that includes a scutellum, which resembles a shield-like cotyledon, all of which are enclosed within the pericarp alongside the persistent endosperm (Fig. 1A). *emb27-1* exhibits a premature arrest in embryo development, whereas the development of the endosperm remains unaffected (Fig. 1B). *EMB27-1* encodes a highly conserved RPS13, a key component of the small subunit of the plastid ribosome. Interestingly, the authors identified a weaker allele, *emb27-2*, exhibiting normal embryo development but displaying albinism and seedling lethality due to its inability to produce functional chloroplasts (Fig. 1C). The authors leveraged both the strong and weak *emb27* mutant alleles to decouple the processes of embryo and chloroplast development.

The divergent phenotypes of the 2 *emb27* alleles led the authors to consider whether a threshold level of *EMB27* expression is required for normal embryo development. To test this hypothesis, they quantified *EMB27* transcript levels in the embryos of both mutants and detected no transcript in *emb27-1* but at least 10% of normal levels in *emb27-2*. This result suggests that *emb27-1* is a null allele while *emb27-2* is of partial loss of function, which could explain the normal embryo development in *emb27-2*. Hence, the authors concluded that *emb27-2* expresses enough EMB27 during embryogenesis to reach a developmental threshold but not enough to sustain chloroplast biogenesis throughout seedling growth.

The authors then tested the correlation between *EMB27* expression and embryo development progression. Maize early embryo development has 3 distinct morphological phases: the ovoid proembryo, the club-shaped transition, and the spade-shaped coleoptilar stage (Vernoud et al. 2005). However, the null mutant *emb27-1* exhibits developmental arrest only at the transition stage. Therefore, the authors crossed *emb27-1*/+ with *emb27-2*/+ to generate trans-heterozygotes that would arrest at different stages of embryo development. As predicted, the trans-heterozygote progeny displayed a spectrum of phenotypes, ranging from albino seedlings to defective embryos with varying severity. The authors quantified *EMB27* transcript levels in these embryos and uncovered a negative correlation between the *EMB27* expression level and the severity of the phenotype. Therefore, this result supports the conclusion that sufficient *EMB27* expression is progressively required throughout maize embryogenesis.

The chloroplast defects in albino *emb27-2* seedlings underscore the role of EMB27 in chloroplast biogenesis. Considering that EMB27 is integral to the ribosomal small subunit, the authors probed the impact of its mutation on the formation of the subunit and the synthesis of chloroplast proteins. They found a significant reduction in the small subunit-associated 16s rRNA, possibly due to its degradation from failing to form a stable complex with the subunit. Additionally, membrane-intrinsic chloroplast proteins, including photosystems and ATP synthase, were undetectable. These findings point to compromised ribosomal subunit assembly and plastid protein translation in the *emb27-2* mutant, thus signifying the essential role of EMB27 in these processes during seedling development in maize.

Because some plastid transcripts require plastid-encoded proteins for splicing, the authors investigated whether this process was affected in both *emb27-1* and *emb27-2* mutants. RT-PCR analysis revealed altered splicing of introns in 4 plastid transcripts in *emb27-1* but not in *emb27-2*. This finding suggests that splicing of plastid transcripts is an early function of EMB27 and potentially contributes to the observed embryogenesis defects. Furthermore, this splicing impairment is shared with other maize mutants exhibiting both plastid translation and embryogenesis defects (Zhang et al. 2013; Xu et al. 2021), supporting the general requirement for specific plastid transcript splicing by plastid-encoded proteins during embryogenesis.

Previous studies on maize have found that mutants with plastid-translation defects exhibit either embryo-defective or albino seedling phenotypes, depending on the genetic background (Zhang et al. 2013; Li et al. 2015; Xu et al. 2021). In the present study, Liu et al. (2024) similarly observed this phenomenon in *emb27* mutants. When heterozygous *emb27-1/+* from a near-isogenic background was crossed with a different inbred line, the hybrid F2 progeny segregated both embryo-defective and albino mutants. This finding suggests that the genetic background influences the expressivity of *EMB27*, consistent with previous reports of genetic modifiers associated with plastid translation in Arabidopsis (Parker et al. 2014). However, Liu et al. (2024) provide further evidence that, unlike in Arabidopsis, the background effect observed in maize does not stem from the disruption of metabolic processes but rather from the loss of a chloroplast signal essential for embryo development (Shen et al. 2013; Zhang et al. 2013).

Liu et al. (2024) offer new insights into the role of a plastid ribosomal protein in maize embryogenesis. Their study decouples the embryo and chloroplast defects observed in *emb27* mutants, providing a valuable roadmap for future investigations into the downstream mechanisms of plastid protein translation. These mechanisms might underpin “retrograde signaling pathways” (Chan et al. 2016) responsible for coordinating a systemic response during embryogenesis. Thus, exploration of these mechanisms could illuminate how chloroplasts act as checkpoints, controlling developmental progression in plant embryos.

## Dive Curated Terms

The following phenotypic, genotypic, and functional terms are of significance to the work described in this paper:

ATP CHEBI: CHEBI:15422

plant embryo AmiGo: PO:0009009
